# The Application of Fe-EDTA and Sodium Silicate Affects the Polyphenols Content in Broccoli and Radish Sprouts

**DOI:** 10.3390/biom11081190

**Published:** 2021-08-11

**Authors:** Henryk Dębski, Wiesław Wiczkowski, Joanna Szablińska-Piernik, Marcin Horbowicz

**Affiliations:** 1 Institute of Biological Sciences, Siedlce University of Natural Sciences and Humanities, Prusa 14, 08-110 Siedlce, Poland; henryk.debski@uph.edu.pl; 2 Department of Chemistry and Biodynamics of Food, Institute of Animal Reproduction and Food Research of the Polish Academy of Sciences, Tuwima 10, 10-748 Olsztyn, Poland; 3 Department of Plant Physiology, Genetics and Biotechnology, University of Warmia and Mazury, Oczapowskiego Str. 1A, 10-719 Olsztyn, Poland; joanna.szablinska@uwm.edu.pl

**Keywords:** sprout, broccoli, radish, elicitor, flavonoid, phenolic acid, antioxidant activity

## Abstract

The effects of elicitors on broccoli (*Brassica oleracea* L. var. *Italica*) and radish (*Raphanus* *sativus* L.) sprouts were evaluated. Seeds and then sprouts were soaked daily for 30 min over 6 days in water (control) or a mixture of FeEDTA and sodium silicate or sodium silicate alone. The contents of the flavonoids and phenolic acids (free, esters, and glycosides) were determined using HPLC-ESI-MS/MS. Phenolic compounds were released from the esters after acid hydrolysis and from the glycosides using alkaline hydrolysis. Quercetin, kaempferol, (‒)-epicatechin, naringenin, apigenin, and luteolin derivatives were found in broccoli and radish sprouts, while derivatives of *iso*-rhamnetin, orientin, and vitexin were not present at measurable levels. The flavonoid contents, especially derivatives of quercetin, were considerably higher in the broccoli sprouts than in the radish sprouts. The quantitatively major phenolic acid content in the sprouts of both species was found to be *p*-hydroxybenzoic acid. Its content in the radish sprouts was several times higher than in the broccoli sprouts. The total flavonoid content of broccoli sprouts was 507–734 µg/g DW, while that of the radish sprouts ranged from 155 µg/g DW to 211 µg/g DW. In contrast, total phenolic acids were higher in radish sprouts, ranging from 11,548 to 13,789 µg/g DW, while in broccoli sprouts, they ranged from 2652 to 4527 µg/g DW, respectively. These differences resulted radish sprouts having higher antioxidant activity compared to broccoli sprouts. The applied elicitors increased the content of the total phenolic acids and the antioxidant activity of radish and broccoli sprouts, while they decreased the level of the total flavonoids in broccoli sprouts.

## 1. Introduction

Seeds of the genus Brassicaceae are often used to produce sprouts and microgreens [1,2,3,4,5,6,7,8,9,10]. Brassicaceae sprouts and microgreens are an excellent source of health-promoting compounds, including glucosinolates and isothiocyanates, polyphenols, minerals, and vitamins [11,12,13]. The radish and broccoli sprouts are mainly examined for their glucosinolate content, which can be further hydrolyzed into isothiocyanates [9,10]. A great deal of research attention has been paid to sulforaphane, whose health properties are well established [14]. Available data have shown that the consumption of sprouts plays an important role in human health and reduces the risk of chronic diseases [10,11,13,15,16].

Flavonoids and hydroxycinnamic acids are the most widespread group of poly-phenolic compounds in plants [17,18,19,20]. The flavonoid biosynthetic pathway is part of a larger phenylpropanoid pathway that also produces such secondary metabolites as phenolic acids, lignins, lignans, and stilbenes [17]. The wide variety of flavonoids is due to the ability of the hydroxyl groups present in them to form glycosidic and ester linkages. The beginning of the pathway is involved in the formation of the hydroxycinnamic acids by l-phenylalanine and malonyl-CoA. The first flavonoids class, the chalcones, is then formed by 4-coumaroyl-CoA. Further, the naringenin chalcone is the precursor for a large number of flavonoids, such as flavonols, flavan-3-ols, isoflavones, and/or flavones [18]. The common biosynthetic precursors of both flavonoids and phenolic acids can be competitive with each other. Therefore, it is important to analyze the content of both major types of phenylpropanoid compounds. Their content is affected by genetic factors and conditions during growth [19]. In general, hydroxycinnamic acids occur in plants as esters or as glycosides, whereas hydroxybenzoic acids are mostly present as glycosides [20]. Generally, Brassicaceae plants contain higher amounts of hydroxycinnamic acid derivatives than flavonoids [21,22]. Specifically, phenolic acid derivatives are predominant in sprouts of these species [2]. The hydroxycinnamic acid derivatives such as *p*-coumaric acid, synapic acid, and ferulic acid are characteristic for these plants [23,24].

Broccoli has been extensively studied for its content of phenolic compounds and has been found to contain gallic acid, caffeic acid, *p*-hydroxybenzoic acid, chlorogenic acid, vanillic acid, syringic acid, ferulic acid, synapic acid, benzoic acid, and *trans*-cinnamic acid as well as kaempferol, quercetin, naringenin, and (+)-catechin [25]. According to Paśko et al. [26], broccoli sprouts contain chlorogenic acid, *p*-coumaric acid, ferulic acid, gentisic acid, and synapic acid along with traces of myricetin, luteolin, quercetin, and apigenin. In the study, synapic, gentisic, and ferulic acids were quantitatively dominant in broccoli sprouts [26,27].

There is much less data available in the literature on the composition of the phenolic compounds in radish sprouts [2,28,29]. It was shown that the main phenolic compounds in radish sprouts were derivatives of sinapic acid [2,28]. In another study, sprouts were shown to contain quercetin, ferulic acid, caffeic acid, and p-coumaric acid as well as their derivatives [29].

To conduct the analysis of a large and diverse group of phenolic compounds is difficult. This is caused by their extraction and purification procedure as well as the lack of available standards and modern equipment. Moreover, the use of retention times for flavonoid identification cannot be the basis for obtaining reliable results. These factors result in highly variable analytical results regarding the presence and the content of phenolic compounds in plants [30,31].

Polyphenols accumulate in response to stress conditions during growth, helping plants to acclimatize to these adverse conditions [32]. Thus, a frequently used method to increase their content is to apply stress factors, which are also chemical compounds (elicitors) or physical factors such as light, its intensity, and its wavelength [14,22,33].

Fortification has been proposed as a way to increase essential microelements that are lacking in the human diet [34,35]. The germination of seeds in the presence of Fe lead to a considerable increase in the content of this element and in the content of phenolic compounds in sprouts [36,37]. Although silicon is an element that is not as important as Fe, its compounds are used to support plant responses to abiotic stresses, but this can lead to increased concentrations of total phenols and flavonoids, as shown in plants grown in the presence of Si [38]. A reduction in antinutrients (trypsin inhibitor, phytic acid, tannin) was also recorded.

To date, chemicals such as methyl jasmonate, sodium selenate or chitosan, or biotic factors that are extracts from *Sacchcaromyces cerevisiae* and *Salix daphnoides* bark have been used to increase the content of phenolic compounds in broccoli sprouts [8,23]. In the case of radish sprouts, chitosan was also used for this purpose [28]. Our previous studies showed that the effect of Fe-EDTA and sodium silicate was dependent on both the species of elicited sprouts and the type of analyzed compounds [37,39]. In general, the elicitors increased the content of phenolic compounds in fenugreek and alfalfa sprouts, whereas they decreased their content in lentil sprouts.

The aim of this study was to investigate the effect of elicitors (Fe-EDTA-Na_2_SiO_3_ or Na_2_SiO_3_ alone) during the germination and growth of broccoli and radish sprouts as a strategy to increase the content of free flavonoids and phenolic acids as well as their esters and glycosides. To the best of our knowledge, there are no published data regarding the effects of the mentioned elicitors on phenolic compounds in sprouts from these species.

## 2. Material and Methods

### 2.1. Plant Material

Broccoli (*Brassica oleracea* L. var. *italica*) and radish (*Raphanus sativus* L.) seeds used for the study were purchased from the Garden Seed and Nursery Stock Company Torseed Co., Torun, Poland. Initially, seeds were disinfected with 70% (*v*/*v*) ethanol for 1 min followed by 2% sodium hypochlorite for 2 min and were rinsed once in 0.01 N HCl and 3 times with distilled water. The disinfected seeds were soaked at 24 °C in distilled water for 4 h and were placed on a layer of sterilized and moist cotton gauze stretched over an open 330 mL jar. Over the next six days, the seeds and sprouts were soaked in distilled water (control) or the elicitor solutions FeEDTA-Na_2_SiO_3_ (trademark Optysil EKO) or Na_2_SiO_3_. Optysil EKO is recommended for use in the early stages of plant development, which activates natural plant defense mechanisms against stress. The most important benefits of its use use in the preparation process are the enhancement of natural plant defense mechanisms and the stimulation of root system development [38]. The elicitor solutions contained sodium silicate with a concentration of 4 mM of Na_2_SiO_3_ or the Optysil, i.e., mixture of Fe-EDTA (0.5 mM) and sodium silicate (4 mM). The soaking lasted 15 min and was conducted twice each day at 9:00 a.m. and at 5:00 p.m. After each treatment, the seeds were placed back on the gauze layer [39].

The sprouts were grown in light conditions of 100–120 µmol/(m^2^⋅s) photosynthetically active radiation (high pressure sodium lamps) at 20 ± 1 °C (day, 16 h) and 16 ± 2 °C (night, 8 h). On the seventh day, the obtained sprouts were collected and cut into 2–3 mm pieces and were freeze-dried in a laboratory freeze dryer (Alpha 1–2 LD plus, Martin Christ, Osterode am Harz, Germany) for 48 h and were used for flavonoid and phenolic acid analyses.

### 2.2. Determination of Phenolic Compounds

Sprout samples were analyzed using HPLC–MS/MS for the determination of various forms of phenolic acids and flavonoids (free, esters, and glycosides). The profile and content of the phenolic acids and flavonoids were determined according to the method of Płatosz et al. [40]. Briefly, a crude extract was obtained from the freeze-dried plant samples with a mixture of methanol, water, and formic acid 80:19.9:0.1 (*v*/*v*/*v*) by extraction, which was repeated five times. The free forms of the phenolic acids and the flavonoids were isolated with diethyl after adjusting the initial extract to pH 2 with 6 M HCl. Next, after free form isolation, esters that were present in the extracts were hydrolyzed with 4 M NaOH. Subsequently, the glycosides that were present in the extracts were hydrolyzed in the residues with 6 M HCl. After each step, the released free forms were isolated with diethyl after adjusting the mixture to pH 2. The obtained ether extracts were evaporated to dryness under nitrogen steam, and the phenolics, both free and released from esters and glycosides, were dissolved in 80% (*v*/*v*) methanol and subjected to HPLC–MS/MS analysis. A HPLC system was equipped with a HALO C18 column (2.7 μm particles, 0.5 × 50 mm, Eksigent, Vaughan, ON, Canada) at 45 °C at a flow rate of 15 μL/min. The elution solvents were A (water/formic acid; 99.05/0.95; *v*/*v*) and B (acetonitrile/formic acid, 99.05/0.95, *v*/*v*). The gradient used was as follows: 5% B for 0.1 min, 5–90% B in 1.9 min, 90% B for 0.5 min, 90–5% B in 0.2 min, and 5% B for 0.3 min. For HPLC–MS/MS analysis, a QTRAP 5500 ion trap mass spectrometer (AB SCIEX, Vaughan, ON, Canada) was applied. Optimal ESI-MS/MS conditions including nitrogen curtain gas (25 L/min), collision gas (9 L/min), ion spray source voltage (−4500 V), temperature (350 °C), nebulizer gas (35 L/min), and turbo gas (30 L/min) were applied. Analyses were conducted in the negative mode by the multiple reaction monitoring (MRM) of selected the ions in the first quadrupole and third quadrupole. The following flavonoids (free, esters, and glycosides) were determined: (‒)-epicatechin, luteolin, orientin, vitexin, apigenin, naringenin, kaempferol, *iso*-rhamnetin, and quercetin (Sigma Aldrich, St. Louis, MO, USA). Moreover, derivatives of the following phenolic acids were analyzed: *p*-hydroxybenzoic, caffeic, sinapic, *p*-coumaric, ferulic, syringic, and chlorogenic (Sigma Aldrich). Details of the LC/MS/MS analysis are shown in Supplemental Table S1. The determination limit was set to 0.5 μg/g DW for the flavonoids and to 1.0 μg/g DW for the phenolic acids. 

### 2.3. Antixidant Activity

The antioxidant activity of broccoli and radish sprout extracts was determined using the free radical 2,2-diphenyl-1-picrylhydrazyl (DPPH^•^) according to the methods of Miller et al. [41]. Methanolic extracts from freeze-dried tissues were incubated at room temperature for 30 min in the dark. After incubation, the absorbance was read at 517 nm using a UV spectrophotometer. The antioxidant activity was calculated on Trolox as equivalent/g dry weight.

### 2.4. Statistics

Analyses of the sprout tissues were performed in three replicates. Analysis of variance (one-way ANOVA) and Tukey’s post hoc test were used to check the significance of the differences. These calculations and the Pearson’s correlation coefficients were performed using Statistica 12PL software (StatSoft, Tulsa, OK, USA). Results in the tables are shown as mean ± standard deviation (SD). Means marked with the same letter are statistically insignificant at *p* > 0.05 (post hoc Tukey’s test). Comparisons were made within each column for each phenolic compound separately. Principal Component Analysis (PCA) was performed in the COVAIN program using MATLAB software (version 2013a, Math Works, Natick, MA, USA) in order to compare each of the elicitors (FeEDTA-Na_2_SiO_3_ and/or Na_2_SiO_3_), treatment profiles of the total flavonoids and phenolic acids in broccoli and radish sprouts were determined.

## 3. Results

The major flavonoid in the broccoli sprouts was quercetin, which was found almost exclusively as glycosides (Table 1). Besides quercetin, kaempferol, (‒)-epicatechin, naringenin, apigenin, and luteolin derivatives were found in these sprouts, whereas *iso*-rhamnetin, orientin, and vitexin were not found at measurable levels. The application of both elicitors resulted in a reduction of quercetin glycosides content as well as the total of its free form, esters, and glycosides. Under the influence of both elicitors there was also a slight decrease in the content of apigenin glycosides and (‒)-epicatechin esters. On the other hand, the application of sodium silicate to broccoli sprouts increased the content of the kaempferol glycosides.

The major phenolic acids found in broccoli sprouts were the *p*-hydroxybenzoic (PHA), ferulic, and sinapic acids, which were present in the free, ester, and glycosidic forms (Table 2). The following acids were present in lower amounts: *p*-coumaric, caffeic, and syringic acids. There was no measurable content of chlorogenic acid. The use FeEDTA-Na_2_SiO_3_ increased the content of the esters, the glycosides, and the total of PHA. Na_2_SiO_3_ alone had a greater effect on the free phenolic acids and their derivatives, as it caused a marked increase in the content the of ester and glycosidic derivatives of almost all of the phenolic acids. The total content of PHA, ferulic, *p*-coumaric, caffeic and syringic acids was also increased, especially under the influence of Na_2_SiO_3_.

Radish sprouts contained lower flavonoid content than broccoli (Table 3). In these sprouts, elicitation decreased the content of kaempferol and quercetin glycosides, but increased the content of their esters. Moreover, Na_2_SiO_3_ increased the content of epicatechin esters. Moreover, radish sprouts contained a very high PHA content, which was 80% present in about in the form of glycosides, about 10–15% in the form of esters, and only about 5% in the free form (Table 4). In addition, ferulic, sinapic, syringic, *p*-coumaric, and caffeic acid esters were also present in relatively high contents, but not chlorogenic acid. The application of FeEDTA-Na_2_SiO_3_ increased the contents of free PHA, its esters and glycosides, and the sum them of all but decreased the contents of the ferulic and *p*-coumaric acid esters. The use of Na_2_SiO_3_ alone increased the levels of the PHA esters and the ferulic and caffeic acids, while it did not affect the content of free PHA, ferulic, and caffeic acids.

Describing the results in Table 5, it should be noted that the flavonoids in broccoli sprouts were primarily in the form of glycosides. The elicitors that were used caused a decrease in the total content of its glycosides as well as in the total content of all of the flavonoids. Phenolic acids in broccoli sprouts occurred in relatively similar contents in free, ester, and glycoside forms but with a slight predominance of ester forms. However, in contrast to flavonoids, the that were elicitors used increased the content of both free phenolic acids and their esters and glycosides. The antioxidant activity of radish sprouts was higher than that of broccoli sprouts. The applied elicitors caused a decrease in the content of the total flavonoids in the broccoli sprouts, but at the same time, they clearly increased the content of the phenolic acids in sprouts of both species as well as their antioxidant activity.

Pearson correlation coefficient analysis showed a low correlation (*r* = 0.5) between antioxidant activity and FeEDTA-Na_2_SiO_3_ treatment in broccoli (Table 6). A high negative correlation (*r* = −0.997) was found between the control and Na_2_SiO_3_ treatment in broccoli. In radish sprouts, very high correlation coefficients (*r* = 0.997 and 0.986) were obtained for both of the elicitors used, FeEDTA-Na_2_SiO_3_ and Na_2_SiO_3_. The correlation coefficients between the total phenolic acids and antioxidant activity are high in the sprouts of both species. In the case of flavonoids, the coefficient was found to be highly negative in broccoli sprouts. This was probably caused by the reduction of the content of these compounds under the influence of the applied elicitors (Table 1 and Table 5).

In radish sprouts, no significant correlation was noted between the total flavonoids and the antioxidant activity, which can be explained by the low content of these compounds in the tissues of this species.

To assess the variation between the samples (control and elicitor-treated), principal component analysis (PCA) was performed, which showed a clear separation between the samples in broccoli sprouts (Figure S1) and radish sprouts (Figure S2). For both species, the discrimination of samples was mainly due to PC1 (contributing about 90% of the variance). In broccoli sprouts, based on the loading plots, the flavonoids quercetin, kaempferol, and apigenin contributed to the separation of the samples (Figure S1A,B), whereas in the phenolic acid group, PHA and sinapic acid were the main contributors (Figure S1C,D). In radish sprouts, kaempferol (Figure S2A,B) played this role in flavonoids, whereas in phenolic acids, caffeic acid and PHA played this role (Figure S2C,D).

## 4. Discussion

The germination process causes major changes in the metabolic composition of plant tissues, one important effect of which is the accumulation of phenolic compounds [1,3,4]. It is also known that phenolic compounds accumulate in response to stress conditions during vegetation, which helps plants to acclimatize to these conditions [8,32,42,43]. On the other hand, a higher content of phenolic compounds is important in the human diet [19,20]. Therefore, some chemical compounds (elicitors) are used to increase the content of flavonoids and phenolic acids in plant tissues [23]. Physical factors such as light, its intensity, and its wavelength are also used for this purpose [14,22,33]. There is a little data in the available literature regarding the effect of sodium silicate and Fe-EDTA on the phenolic compound profile of plants. Hence, the studies that have been undertaken, which are described in our previous and present work.

In Brassicaeae vegetables, derivatives of flavonols and hydroxycinnamic acids are characteristic [21,22,23,24,25,26,27,28,29]. Broccoli tissues contain higher levels of quercetin than kaempferol, which has been confirmed in our study although there is great variation in their content among cultivars [10,13]. Moreover, in broccoli sprouts, gallic acid, caffeic acid, PHA, chlorogenic acid, vanillic acid, syringic acid, ferulic acid, synapic acid, benzoic acid, *trans*-cinnamic acid were found [26,27]. Among them synapic acid, gentisic acid and ferulic acid were quantitatively dominant. In contrast, Gawlik-Dziki et al. [8] demonstrated the presence of (+)-catechin, quercetin, kaempferol, as well as PHA, chlorogenic acid, caffeic acid, syringic acid, *p*-coumaric acid, ferulic acid, and sinapic acid. These last-cited data are similar to the results of our study, except for chlorogenic acid and (+)-catechin in broccoli sprouts (Table 1 and Table 2).

Literature data on the content of phenolic compounds in radish sprouts are relatively limited compared to broccoli sprouts [2,9,28,29]. According to Li and Zhu [29], radish sprouts contain quercetin, ferulic acid, caffeic acid and *p*-coumaric, and their derivatives, and this was confirmed in our study. In other studies, it was shown that major phenolic compounds in radish sprouts were the derivatives of sinapic acid [2,28]. In our study, the content of synapic acid was much lower than that of PHA or ferulic acid (Table 4). According to a study by Pająk et al. [2], radish sprouts contained low levels of free flavonoids, their derivatives, and free phenolic acids, and these acids occur mainly as glycosides. Our results partly confirm these data (Table 3 and Table 4). Flavonoids were present in very small amounts, and the major phenolic acid was PHA, which was found predominantly in its glycosidic form. The other acids were mainly found in their ester forms.

Discrepancies between the phenolic composition in our study and those previously published may be due to fact that a number of environmental factors modify the secondary metabolite accumulation in plants [44]. Of these, germination and sprout growth conditions and the method of elicitation appear to be the most important. Another significant factor is the genetic diversity of plant objects [45]. Furthermore, the use of elicitors to increase the nutritional value of sprouts does not always lead to this treatment having predictable effects [46]. Added to this is the influence of the method used to isolate phenolic compounds from sprouts and the technique used to analyze them [47]. In conclusion, all of the factors and circumstances mentioned above make each experiment in this field different from those previously described.

Treatment with silicon compounds is mainly used for studies on its role in abiotic and biotic plant stresses [42,43]. Previously, evidence was shown that silicon enhanced the level of rhamnetin in cucumber plants [48]. The evaluation and discussion of the results obtained in the present study compared to those of others is difficult due to the lack of information regarding the effects of the investigated elicitors on sprouts or adult plants of the genus Brassicaceae. Our previous studies showed that elicitation with FeEDTA-Na_2_SiO_3_ affected flavonoids and phenolic acids in sprouts of other species in various ways [37,39]. Thus, the content of flavonoids in buckwheat sprouts treated with these elicitors decreased [37]. Moreover, FeEDTA-Na_2_SiO_3_ decreased the level of free (‒)-epicatechin and enhanced the content of its esters in fenugreek and alfalfa sprouts [39]. Moreover, elicitors decreased the content of quercetin glycosides in lentil and fenugreek sprouts. Similarly, the results of the current study show that the applied elicitors reduced the content of the main flavonoid in the quercetin glycosides of broccoli sprouts. In addition, they also slightly increased the content of kaempferol glycosides in both broccoli and radish sprouts. Additionally, the elicitors that were investigated had a similar effect as in the previous study on the contents of phenolic acids [39]. The content of most phenolic acids in broccoli sprouts increased, while in radish sprouts, a marked increase occurred for *p*-hydroxybenzoic acid and caffeic acid.

The antioxidant activity of radish sprouts was higher than that of broccoli sprouts, which is a confirmation of previous results [2,11,13]. The high antioxidant activity of radish sprouts is probably due to the fact that their phenolic acid content is several time higher than that of broccoli sprouts. Among the elicitors that were used in the study, sodium silicate was more effective in enhancing the antioxidant activity than the mixture with FeEDTA. Unfortunately, the small number of data used to do the correlation calculations between the applied elicitors and antioxidant activity does not allow us to use the obtained coefficients to make clear conclusions. We can only formulate tendencies that indicate that the elicitors used in this study strongly promote the elevation of antioxidant activity in radish sprouts. In contrast, a negative effect of Na_2_SiO_3_ and a slight positive effect of FeEDTA-Na_2_SiO_3_ on antioxidant activity can be observed in broccoli sprouts.

Iron is microelement that is essential for human health, and its dietary deficiency affects over a quarter of the world population, leading to a primary global public health problem [49,50]. A solution to this problem could be biofortification during seed sprouting [37]. A study on buckwheat sprouts showed a 5-fold and 2-fold increase in Fe and Si content after the application of Fe-EDTA and sodium silicate, respectively [37]. However, sprout germination in the presence of Fe can lead to a reduction in the fresh yield of broccoli and radish sprouts [36]. Furthermore, using iron compounds only during seed imbibition may not increase the iron content and the level of phenolic compounds in the sprouts [34].

## 5. Conclusions

In general, the results of the present study indicate that in the sprouts of both species, flavonoids mainly occur in the form of esters and glycosides, and their free forms are present at low levels, i.e., below 5 µg/g DW. The major flavonoids in broccoli sprouts were quercetin glycosides and were kaempferol glycosides in radish sprouts. In radish sprouts, the flavonoid content was considerably lower than in broccoli sprouts. In contrast, radish sprouts contained significantly higher levels of phenolic acids than broccoli sprouts, especially *p*-hydroxybenzoic acid, which was also the major phenolic acid in the sprouts of both species. The phenolic acids of broccoli and radish sprouts occur as free, esters, and glycosides, and their total contents were many fold higher than those of flavonoids.

The elicitors used and the conditions under which the sprouts were obtained reduced the total flavonoid content of broccoli sprouts. However, this was accompanied by an increase in the content of phenolic acid derivatives. As a final result, the total content of all of the phenolic compounds also increased. In the case of radish sprouts, the elicitors that were used increased the levels of the quantitatively major *p*-hydroxybenzoic acid as well as caffeic acid.

As a result of the increase in the total content of the analyzed phenolic compounds in the sprouts of both species, an increase in the antioxidant activity was demonstrated. The antioxidant activity of the radish sprouts was higher than that of the broccoli sprouts, which is probably due to the higher total content of phenolic compounds in these sprouts.

## Figures and Tables

**Table 1:** The content (µg/g DW) of flavonoids (free, released from ester and *O*-glycoside forms, and in total) in broccoli sprouts treated with elicitors during growth.

Treatment	Free	Esters	Glycosides	Total
Quercetin				
Control	2.1 ± 0.2 ^a^	9.9 ± 0.3 ^a^	516 ± 8.2 ^a^	528 ± 9.0 ^a^
FeEDTA-Na_2_SiO_3_	2.4 ± 0.2 ^a^	4.9 ± 0.3 ^b^	407 ± 7.7 ^b^	414 ± 7.5 ^b^
Na_2_SiO_3_	nd	5.2 ± 0.2 ^b^	325 ± 5.6 ^c^	330 ± 5.8 ^c^
Kaempferol				
Control	nd	1.3 ± 0.2 ^a^	58.8 ± 1.4 ^b^	60.1 ± 1.6 ^b^
FeEDTA-Na_2_SiO_3_	nd	1.0 ± 0.1 ^a^	61.2 ± 1.3 ^b^	62.2 ± 1.5 ^b^
Na_2_SiO_3_	nd	1.2 ± 0.2 ^a^	96.5 ± 2.5 ^a^	97.7 ± 2.7 ^a^
Naringenin				
Control	1.9 ± 0.1 ^a^	Nd	6.9 ± 0.2 ^b^	8.8 ± 0.5 ^b^
FeEDTA-Na_2_SiO_3_	1.0 ± 0.3 ^a^	Nd	6.5 ± 0.3 ^b^	7.5 ± 0.6 ^b^
Na_2_SiO_3_	1.1 ± 0.3 ^a^	Nd	13.6 ± 0.3 ^a^	14.7 ± 0.6 ^a^
(‒)-Epicatechin				
Control	nd	48.3 ± 0.8 ^a^	nd	48.3 ± 0.8 ^a^
FeEDTA-Na_2_SiO_3_	nd	39.4 ± 0.6 ^b^	nd	39.4 ± 0.6 ^b^
Na_2_SiO_3_	nd	37.6 ± 0.7 ^b^	nd	37.6 ± 0.7 ^b^
Apigenin				
Control	nd	2.9 ± 0.4 ^a^	38.8 ± 1.6 ^a^	41.7 ± 2.0 ^a^
FeEDTA-Na_2_SiO_3_	nd	Nd	15.5 ± 2.0 ^b^	15.5 ± 2.0 ^b^
Na_2_SiO_3_	nd	1.1 ± 0.3 ^a^	23.7 ± 1.8 ^b^	24.8 ± 2.1 ^b^
Luteolin				
Control	nd	1.0 ± 0.2 ^a^	1.0 ± 0.1 ^a^	2.0 ± 0.3 ^a^
FeEDTA-Na_2_SiO_3_	nd	1.0 ± 0.1 ^a^	1.0 ± 0.2 ^a^	2.0 ± 0.3 ^a^
Na_2_SiO_3_	nd	Nd	2.1 ± 0.3 ^a^	2.1 ± 0.3 ^a^

Abbreviations: FeEDTA-Na_2_SiO_3_, FeEDTA chelate and sodium silicate; nd, not detected; DW, dry weight. Mean results ± SD followed by the same letter within the same column and calculated for each flavonoid separately were not significantly different (*p* < 0.05) according to Tukey’s test.

**Table 2:** The content (µg/g DW) of phenolic acids (free, released from ester and O-glycoside forms, and in total) in broccoli sprouts (µg/g DW) treated with elicitors during growth.

Treatment	Free	Esters	Glycosides	Total
*p*-Hydroxybenzoic (PHA)				
Control	789 ± 22.3 ^b^	236 ± 10.4 ^b^	596 ± 24.1 ^c^	1621 ± 54.8 ^c^
FeEDTA-Na_2_SiO_3_	772 ± 18.3 ^b^	385 ± 11.2 ^a^	943 ± 26.7 ^b^	2050 ± 56.2 ^b^
Na_2_SiO_3_	1214 ± 26.7 ^a^	391 ± 19.1 ^a^	1215 ± 38.2 ^a^	2820 ± 84.1 ^a^
Ferulic				
Control	30.9 ± 6.1 ^b^	236 ± 14.2 ^b^	5.5 ± 0.5 ^a^	272 ± 20.8 ^b^
FeEDTA-Na_2_SiO_3_	77.1 ± 8.4 ^a^	297 ± 16.8 ^b^	7.3 ± 1.1 ^a^	381 ± 26.3 ^b^
Na_2_SiO_3_	32.4 ± 7.2 ^b^	570 ± 19.1 ^a^	8.4 ± 0.9 ^a^	611 ± 27.1 ^a^
Sinapic				
Control	97.5 ± 9.1 ^a^	274 ± 10.4 ^a^	53.8 ± 8.3 ^a^	425 ± 27.3 ^a^
FeEDTA-Na_2_SiO_3_	133 ± 14.3 ^a^	305 ± 14.7 ^a^	66.6 ± 9.1 ^a^	505 ± 38.1 ^a^
Na_2_SiO_3_	35.3 ± 5.4 ^b^	324 ± 17.7 ^a^	56.8 ± 7.3 ^a^	416 ± 30.4 ^a^
*p*-Coumaric				
Control	4.1 ± 0.3 ^a^	83.3 ± 9.1 ^b^	3.5 ± 0.1 ^a^	90.9 ± 9.5 ^b^
FeEDTA-Na_2_SiO_3_	2.2 ± 0.4 ^a^	104 ± 6.3 ^b^	3.5 ± 0.2 ^a^	110 ± 6.9 ^b^
Na_2_SiO_3_	5.3 ± 0.5 ^a^	164 ± 12.2 ^a^	3.1 ± 0.1 ^a^	172 ± 12.7 ^a^
Caffeic				
Control	nd	83.9 ± 6.8 ^b^	6.7 ± 0.1 ^b^	90.6 ± 6.9 ^b^
FeEDTA-Na_2_SiO_3_	nd	103 ± 9.1 ^b^	9.4 ± 0.4 ^a^	112 ± 9.5 ^b^
Na_2_SiO_3_	nd	253 ± 12.9 ^a^	11.6 ± 0.6 ^a^	265 ± 13.5 ^a^
Syringic				
Control	nd	151 ± 9.2 ^c^	nd	151 ± 9.2 ^c^
FeEDTA-Na_2_SiO_3_	nd	193 ± 8.4 ^b^	nd	193 ± 8.4 ^b^
Na_2_SiO_3_	nd	243 ± 12.4 ^a^	nd	243 ± 12.4 ^a^

Abbreviations: FeEDTA-Na_2_SiO_3_, FeEDTA chelate and sodium silicate; nd, not detected; DW, dry weight. Mean results ± SD followed by the same letter within the same column and calculated for each phenolic acid separately were not significantly different (*p* < 0.05) according to Tukey’s test.

**Table 3:** The content (µg/g DW) of flavonoids (free, released from ester and *O*-glycoside forms, and in total) in radish sprouts treated with elicitors during growth.

Treatment	Free	Esters	Glycosides	Total
Quercetin				
Control	nd	3.1 ± 0.2 ^b^	16.8 ± 0.7 ^a^	19.9 ± 0.9 ^a^
FeEDTA-Na_2_SiO_3_	nd	5.9 ± 0.3 ^b^	7.3 ± 0.3 ^b^	13.2 ± 0.6 ^b^
Na_2_SiO_3_	nd	14.7 ± 0.5 ^a^	6.1 ± 0.2 ^b^	20.8 ± 0.7 ^a^
Kaempferol				
Control	nd	10.6 ± 0.3 ^c^	66.1 ± 2.2 ^a^	76.7 ± 2.5 ^b^
FeEDTA-Na_2_SiO_3_	nd	49.2 ± 1.3 ^b^	8.6 ± 0.6 ^b^	57.8 ± 1.9 ^c^
Na_2_SiO_3_	nd	87.0 ± 3.3 ^a^	14.2 ± 1.5 ^b^	101.2 ± 4.8 ^a^
Naringenin				
Control	3.3 ± 0.3 ^a^	nd	2.2 ± 0.3 ^a^	5.3 ± 0.6 ^a^
FeEDTA-Na_2_SiO_3_	1.4 ± 0.4 ^a^	1.1 ± 0.2 ^a^	1.6 ± 0.1 ^a^	4.1 ± 0.7 ^a^
Na_2_SiO_3_	nd	1.7 ± 0.4 ^a^	nd	1.7 ± 0.4 ^a^
Apigenin				
Control	1.1 ± 0.3 ^a^	2.4 ± 0.4 ^a^	17.5 ± 0.5 ^a^	21.0 ± 1.3 ^a^
FeEDTA-Na_2_SiO_3_	4.3 ± 0.7 ^a^	6.2 ± 0.6 ^a^	17.2 ± 0.5 ^a^	27.7 ± 1.8 ^a^
Na_2_SiO_3_	1.0 ± 0.3 ^a^	5.2 ± 0.5 ^a^	16.8 ± 0.6 ^a^	23.0 ± 1.4 ^a^
Luteolin				
Control	nd	1.2 ± 0.2 ^c^	nd	1.2 ± 0.2 ^c^
FeEDTA-Na_2_SiO_3_	nd	4.9 ± 0.3 ^b^	nd	4.9 ± 0.3 ^b^
Na_2_SiO_3_	nd	9.7 ± 0.8 ^a^	2.4 ± 0.2 ^a^	12.1 ± 1.0 ^a^
(‒)-Epicatechin				
Control	nd	30.7 ± 1.4 ^b^	nd	30.7 ± 1.4 ^b^
FeEDTA-Na_2_SiO_3_	nd	38.2 ± 1.8 ^b^	nd	38.2 ± 1.8 ^b^
Na_2_SiO_3_	nd	51.5 ± 2.4 ^a^	nd	51.5 ± 2.4 ^a^

Abbreviations: FeEDTA-Na_2_SiO_3_, FeEDTA chelate and sodium silicate; nd, not detected; DW, dry weight. Mean results ± SD followed by the same letter within the same column and calculated for each flavonoid separately were not significantly different (*p* < 0.05) according to Tukey’s test.

**Table 4:** The content (µg/g DW) of phenolic acids (free, released from ester and O-glycoside forms, and in total) in radish sprouts (µg/g DW) treated with elicitors during growth.

Treatment	Free	Esters	Glycosides	Total
*p*-Hydroxybenzoic (PHA)				
Control	310 ± 6.9 ^b^	961 ± 21.2 ^b^	7614 ± 32.2 ^c^	8885 ± 60.2 ^c^
FeEDTA-Na_2_SiO_3_	424 ± 8.7 ^a^	1933 ± 26.8 ^a^	8670 ± 26.8 ^a^	11,027 ± 62.3 ^a^
Na_2_SiO_3_	291 ± 5.8 ^b^	1854 ± 36.1 ^a^	8457 ± 36.1 ^b^	10,602 ± 79.1 ^b^
Ferulic				
Control	386 ± 6.9 ^b^	647 ± 9.7 ^b^	9.7 ± 0.6 ^a^	1043 ± 16.9 ^b^
FeEDTA-Na_2_SiO_3_	452 ± 8.2 ^a^	468 ± 8.2 ^c^	8.1 ± 0.7 ^a^	928 ± 17.1 ^c^
Na_2_SiO_3_	361 ± 6.5 ^b^	779 ± 10.5 ^a^	7.6 ± 0.5 ^a^	1148 ± 17.5 ^a^
Sinapic				
Control	177 ± 6.2 ^a^	336 ± 8.2 ^a^	57.3 ± 5.2 ^a^	570 ± 19.6 ^a^
FeEDTA-Na_2_SiO_3_	178 ± 4.5 ^a^	371 ± 9.7 ^a^	27.9 ± 4.4 ^b^	577 ± 18.6 ^a^
Na_2_SiO_3_	124 ± 5.1 ^b^	381 ± 10.1 ^a^	28.1 ± 3.2 ^b^	532 ± 18.4 ^a^
Syringic				
Control	nd	291 ± 8.5 ^ab^	nd	291 ± 8.5 ^ab^
FeEDTA-Na_2_SiO_3_	nd	264 ± 7.5 ^b^	nd	264 ± 7.5 ^b^
Na_2_SiO_3_	nd	317 ± 9.9 ^a^	nd	317 ± 9.9 ^a^
*p*-Coumaric				
Control	6.2 ± 0.7 ^b^	284 ± 10.1 ^a^	2.1 ± 0.2 ^a^	292 ± 11.1 ^a^
FeEDTA-Na_2_SiO_3_	10.8 ± 1.2 ^ab^	176 ± 8.3 ^b^	2.1 ± 0.2 ^a^	189 ± 9.7 ^b^
Na_2_SiO_3_	16.2 ± 1.4 ^a^	255 ± 9.3 ^a^	2.6 ± 0.3 ^a^	274 ± 11.0 ^a^
Caffeic				
Control	6.4 ± 0.3 ^a^	302 ± 8.5 ^b^	2.4 ± 0.4 ^b^	311 ± 9.2 ^b^
FeEDTA-Na_2_SiO_3_	5.5 ± 0.5 ^a^	305 ± 9.2 ^b^	6.1 ± 0.7 ^a^	317 ± 10.4 ^b^
Na_2_SiO_3_	8.9 ± 0.7 ^a^	687 ± 12.4 ^a^	8.5 ± 0.8 ^a^	704 ± 13.9 ^a^

Abbreviations: FeEDTA-Na_2_SiO_3_, FeEDTA chelate and sodium silicate; nd, not detected; DW, dry weight. Mean results ± SD followed by the same letter within the same column and calculated for each phenolic acid separately were not significantly different (*p* < 0.05) according to Tukey’s test.

**Table 5:** The total content of phenolic acids and flavonoids (µg/g DW) and antioxidant capacity (Trolox equivalent, µM/g DW) in broccoli and radish sprouts treated with elicitors during growth.

Treatment	Free	Esters	Glycosides	Total
Broccoli, total flavonoids				
Control	4.0 ± 0.7 ^a^	63.4 ± 4.5 ^a^	667 ± 12 ^a^	734 ± 18 ^a^
FeEDTA-Na_2_SiO_3_	3.4 ± 0.9 ^a^	46.3 ± 2.1 ^b^	491 ± 14 ^b^	541 ± 17 ^b^
Na_2_SiO_3_	1.1 ± 1.0 ^a^	45.1 ± 2.3 ^b^	461 ± 17 ^b^	507 ± 20 ^b^
Broccoli, total phenolic acids				
Control	922 ± 22 ^b^	1064 ± 18 ^c^	666 ± 13 ^c^	2652 ± 53 ^b^
FeEDTA-Na_2_SiO_3_	984 ± 19 ^b^	1387 ± 24 ^b^	1030 ± 22 ^b^	3401 ± 65 ^b^
Na_2_SiO_3_	1287 ± 12 ^a^	1945 ± 23 ^a^	1295 ± 24 ^a^	4527 ± 59 ^a^
Broccoli, total flavonoids and phenolic acids				
Control	926 ± 22 ^b^	1127 ± 22 ^c^	1333 ± 25 ^c^	3386 ± 71 ^c^
FeEDTA-Na_2_SiO_3_	987 ± 19 ^b^	1433 ± 26 ^b^	1521 ± 34 ^b^	3941 ± 82 ^b^
Na_2_SiO_3_	1288 ± 12 ^a^	1990 ± 26 ^a^	1756 ± 42 ^a^	5034 ± 80 ^a^
	Broccoli, antioxidant activity			
Control	33.5 ± 2.9 ^b^			
FeEDTA-Na_2_SiO_3_	43.3 ± 1.9 ^a^			
Na_2_SiO_3_	47.4 ± 1.3 ^a^			
Radish, total flavonoids				
Control	4.4 ± 1.2 ^a^	48.0 ± 3.5 ^c^	103 ± 10 ^a^	155 ± 15 ^a^
FeEDTA-Na_2_SiO_3_	5.4 ± 1.5 ^a^	106 ± 10 ^b^	34.7 ± 3.5 ^b^	146 ± 15 ^a^
Na_2_SiO_3_	1.0 ± 0.5 ^a^	170 ± 14 ^a^	39.5 ± 4.5 ^b^	211 ± 18 ^a^
Radish, total phenolic acids				
Control	886 ± 22 ^b^	2821 ± 51 ^c^	7686 ± 63 ^c^	11,393 ± 133 ^b^
FeEDTA-Na_2_SiO_3_	1070 ± 27 ^a^	3517 ± 53 ^b^	8714 ± 70 ^b^	13,301 ± 140 ^a^
Na_2_SiO_3_	801 ± 24 ^b^	4273 ± 43 ^a^	8504 ± 67 ^a^	13,578 ± 133 ^a^
Radish, total flavonoids and phenolic acids				
Control	890 ± 23 ^b^	2869 ± 54 ^c^	7789 ± 73 ^b^	11,548 ± 148 ^b^
FeEDTA-Na_2_SiO_3_	1075 ± 28 ^a^	3623 ± 63 ^b^	8749 ± 74 ^a^	13,447 ± 155 ^a^
Na_2_SiO_3_	802 ± 25 ^b^	4443 ± 67 ^a^	8544 ± 71 ^a^	13,789 ± 151 ^a^
	Radish, antioxidant activity			
Control	49.5 ± 1.9 ^b^			
FeEDTA-Na_2_SiO_3_	63.1 ± 1.1 ^a^			
Na_2_SiO_3_	58.7 ± 1.6 ^a^			

Abbreviations: FeEDTA-Na_2_SiO_3_, FeEDTA chelate and sodium silicate; nd, not detected; DW, dry weight. Mean results ± SD followed by the same letter within the same column and calculated for each phenolic compounds and antioxidant capacity separately were not significantly different (*p* < 0.05) according to Tukey’s test.

**Table 6:** Pearson’s correlation coefficients of antioxidant activity among control and used elicitors as well as between total flavonoids and phenolic acids and antioxidant activity in broccoli and radish sprouts.

Calculated Correlation	Broccoli	Radish
Control vs. FeEDTA-Na_2_SiO_3_	0.500	0.997
Control vs. Na_2_SiO_3_	−0.997	0.986
Total flavonoids vs. antioxidant activity	−0.987	0.077
Total phenolic acids vs. antioxidant activity	0.951	0.911

## Data Availability

The data presented in this study are available in this article.

## Supplementary Materials

The following is available online at https://www.mdpi.com/article/10.3390/biom11081190/s1, Table S1: The conditions for HPLC/MS/MS analysis of phenolic acids and flavonoids identified in broccoli and radish sprouts. Figure S1. Score scatter plots of PCA of flavonoids (A) and phenolic acids (C) profiles for control and applied elicitors (FeEDTA-Na_2_SiO_3_ or Na_2_SiO_3_) in broccoli sprouts and PCA loadings plots (B,D), respectively. Figure S2. Score scatter plots of PCA of flavonoids (A) and phenolic acids (C) profiles for control and applied elicitors (FeEDTA-Na_2_SiO_3_ or Na_2_SiO_3_) in radish sprouts and PCA loadings plots (B,D), respectively.
