# Supplementary material for: The Application of Fe-EDTA and Sodium Silicate Affects the Polyphenols Content in Broccoli and Radish Sprouts
Source: Biomolecules. 2021 Aug 11;11(8):1190. doi: 10.3390/biom11081190 (PMC8392375; doi:10.3390/biom11081190)
Supplement: Supplementary file 1 [file biomolecules-11-01190-s001.zip › biomolecules-1267263-supplementary.pdf]

Supplementary files

Splementary Table S1. The conditions for HPLC/MS/MS analysis of phenolic acids and flavonoids identified in broccoli and radish sprouts

Analysed compound	[M] (m/z)	MS/MS (m/z)
	Phenolic acids	
Chlorogenic acid	353	191/179
Caffeic acid	179	135/107
Syringic acid	197	182/153
Sianapic acid	223	208/179/164
Ferulic acid	193	178/134
p-Coumaric acid	163	119/93
p-Hydroxybenzoic acid (PHA)	137	98/93
	Flavonoids	
Vitexin	431	321/311/283
(–)-Epicatechin	289	245/203/109
Luteolin	285	151/133
Quercetin	301	179/151
Apigenin	269	225/151/117
Kaempferol	285	185/93
Orientin	447	357/339/296

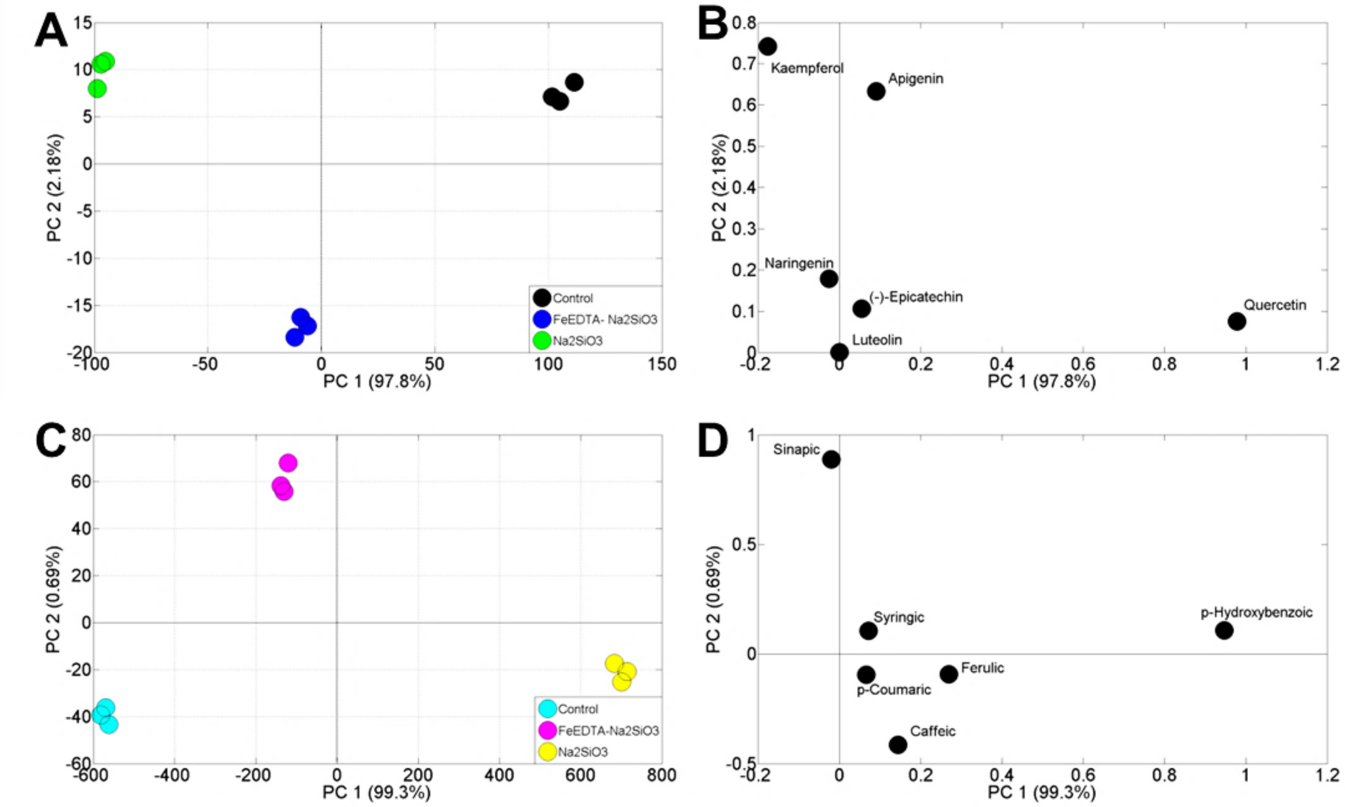

Figure S1. Score scatter plots of PCA of flavonoids (**A**) and phenolic acids (**C**) profiles for control and applied elicitors (FeEDTA-Na_2SiO_3 or Na_2SiO_3) in broccoli sprouts and PCA loadings plots (**B, D**), respectively.

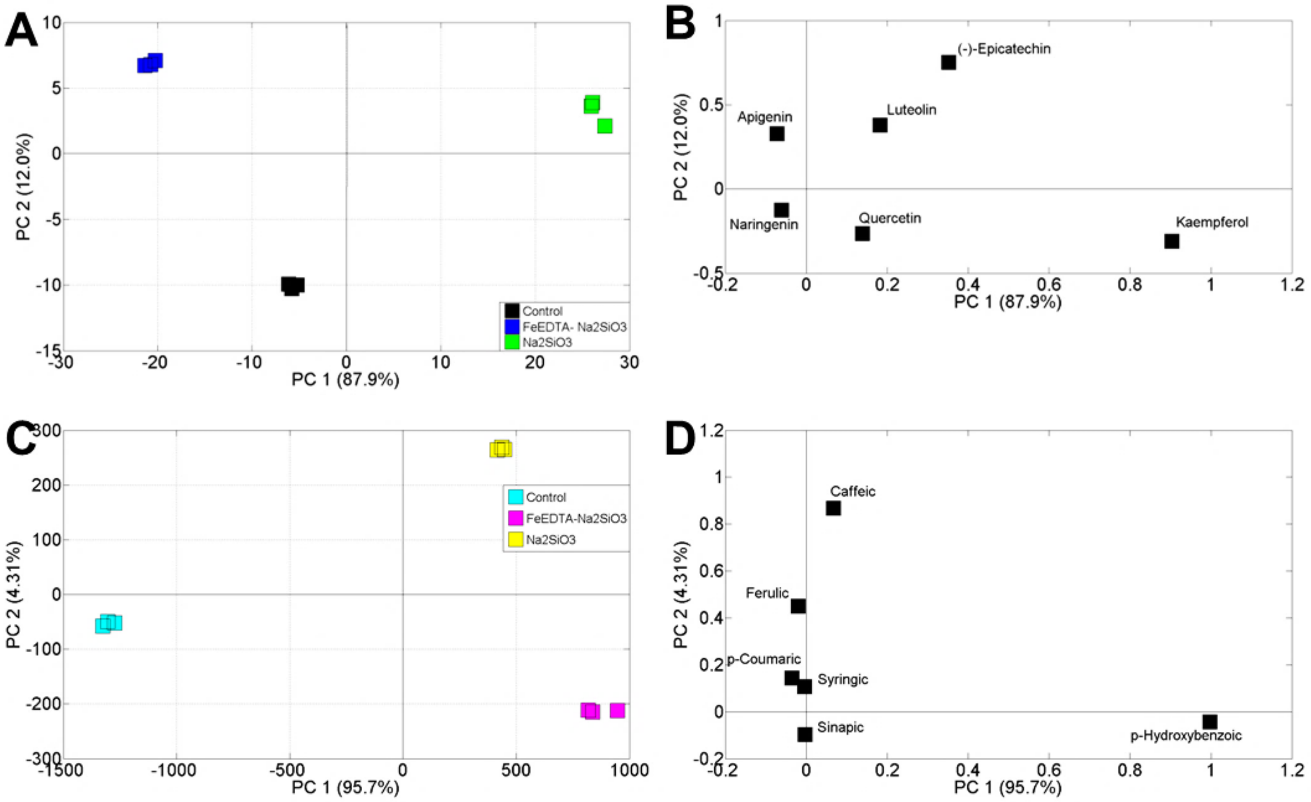

Figure S2. Score scatter plots of PCA of flavonoids (**A**) and phenolic acids (**C**) profiles for control and applied elicitors (FeEDTA-Na_2SiO_3 or Na_2SiO_3) in radish sprouts and PCA loadings plots (**B, D**), respectively.
